# Successful Treatment of Severe Purpura Fulminans With Anakinra

**DOI:** 10.1111/pde.70034

**Published:** 2025-10-08

**Authors:** Francesco Zulian, Luca Spiezia, Antonio Amabile, Francesca Tirelli, Alessandra Meneghel

**Affiliations:** ^1^ Department of Women's and Children's Health Padova University Hospital Padova Italy; ^2^ Department of Medicine Padova University Hospital Padova Italy; ^3^ Clinic of Plastic Surgery University Hospital of Padua Padova Italy

**Keywords:** biological agent, disseminated intravascular coagulation (DIC), skin necrosis, therapy

## Abstract

Purpura fulminans (PF) is a rare, often fatal pediatric condition characterized by intravascular thrombosis and hemorrhagic infarction of the skin. A timely diagnosis and treatment are paramount to prevent the involvement of internal organs, causing disseminated intravascular coagulation and gangrene of the extremities. The management of PF requires a comprehensive approach, including the treatment of the underlying infection, anticoagulation, and anti‐inflammatory therapy. Herein we present the case of a child with severe PF successfully treated with anakinra, an anti‐IL1‐receptor monoclonal antibody.

## 1 Introduction

Purpura fulminans (PF) is an acute and often progressive disease characterized by sudden onset of purpuric lesions, especially on the extremities, that rapidly evolve toward the formation of necrotic blisters associated with necrosis, and even immunocompetent adults can develop life‐threatening complications [1, 2]. It is a rare pediatric emergency due to a microangiopathic vasculitis that causes thrombosis in the dermis. A prompt diagnosis and effective treatment are mandatory. Otherwise, the process can extend to internal organs, causing disseminated thrombosis with ischemia of the distal extremities. Limb amputation is reported in 81% of adult cases in one meta‐analysis and death in 10%–15% [1, 2, 3, 4].

Three clinical subtypes of PF are commonly recognized: neonatal PF caused by a congenital deficiency of protein C and protein S [5], idiopathic or post‐infectious PF that generally involves the skin such as chickenpox or scarlet fever [5, 6] and the most common subtype, acute infectious PF, a well‐known complication of meningococcal and pneumococcal septicemia [7]. Treatment of PF consists of supportive therapy with adequate hydration and maintenance of vital functions, treatment of the underlying infection, and early anticoagulant therapy [3] sometimes with the use of protein‐C concentrate [8].

For the post‐infectious forms, corticosteroids are also recommended, which, however, are not always fully effective and are also contraindicated due to their procoagulant activity [9]. Rapid diagnosis and adequate treatment are therefore essential to early stop the thrombotic process that, from the superficial areas of the skin, can rapidly disseminate to internal vessels and organs. No international experience does exist on the use of biological agents for the treatment of PF. Herein, we present the case of a very young child with severe PF successfully treated with anakinra, a biotechnological anti IL‐1 monoclonal antibody.

## 2 Case Report

An 11‐month‐old Caucasian boy was admitted to the pediatric Emergency Department of Padova University Hospital for high‐grade fever, as well as rapidly progressing purpuric lesions and bilateral cyanosis of the distal extremities in the preceding 24 h. The patient's parents reported that, ten days prior, he had developed an onset of fever (39°C–40.5°C), sore throat, and rhinitis. At that time, he was taken to the local emergency department and diagnosed with a viral infection. The day before admission, purpuric lesions appeared on the distal extremities and progressively worsened.

**FIGURE 1:**
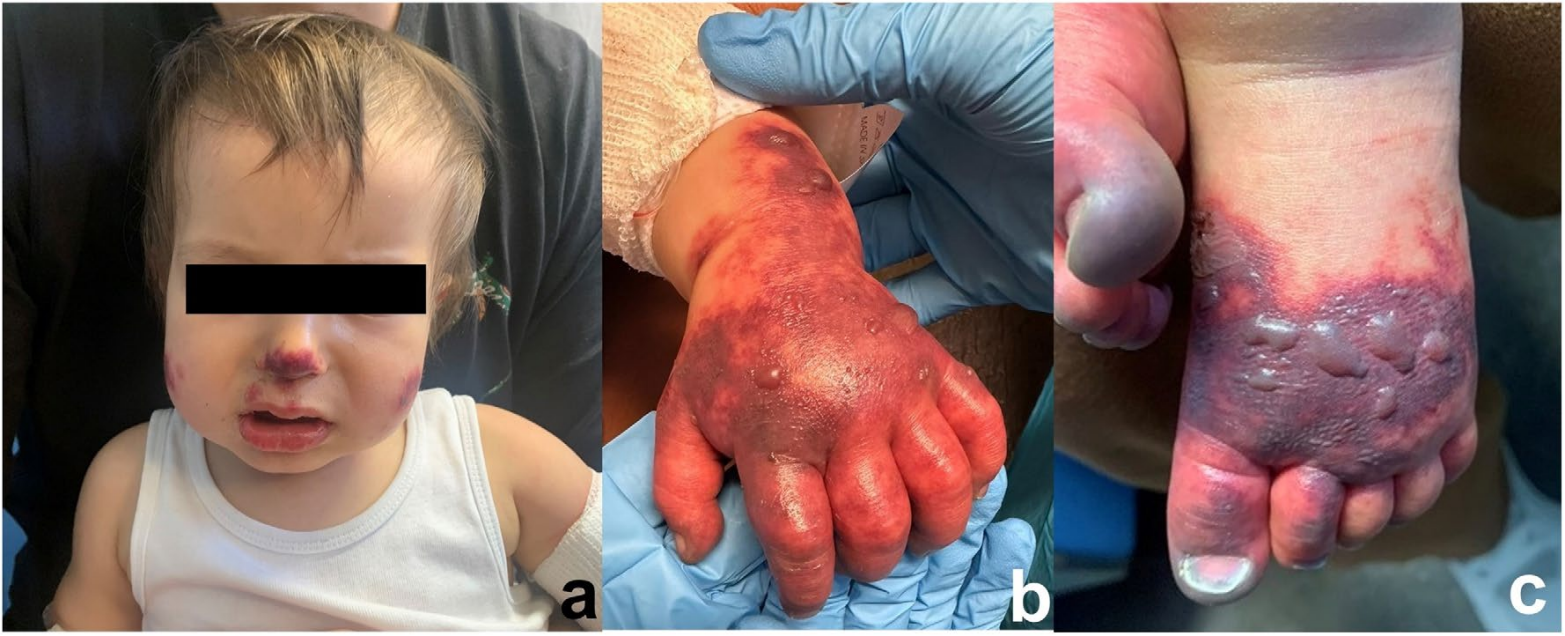
Clinical picture on admission: Grimacing patient in pain with purpuric lesions on nose, lips and cheeks (a), confluent purpuric lesions with blisters and cyanosis of the distal extremities (b, c).

Upon admission, physical examination showed a febrile patient (39.5°C–40°C), irritable and in pain. Purpuric lesions were present on upper and lower extremities, auricles, and cheeks (Figure 1a). The extremities were edematous, hyperemic‐cyanotic, cold, and very painful to touch. Hemorrhagic and vesicular lesions were noted over the dorsum of the hands and feet (Figure 1b,c). No ischemic ulcers were present. Peripheral pulses were present and symmetric. Examination of other systems was normal. The vital signs remained normal with no need for vasoactive drugs.

Laboratory tests were as follows: WBC 17,500/mm^3^ (66% neutrophils), hemoglobin 91 g/L, platelet count 888,000/mm^3^, ESR 46 mm/h, CRP 113 mg/L, INR 0.93, D dimer 26,600 μg/L FEU, fibrinogen 3.93 g/L. Liver, renal, and cardiac function tests were all normal. Results of testing for cold agglutinins, antinuclear antibodies, and coagulation panel including protein‐C, protein‐S, anticardiolipin antibodies, and LAC were all normal. Serum PCR and serology (IgM) assays confirmed an adenovirus infection while other common pathogens ( *Mycoplasma pneumoniae* , SarsCov2, respiratory syncytial virus, enterovirus, influenza A and B, parvovirus B19, Epstein Barr virus, cytomegalovirus, hepatitis A, B, or C) were excluded. The only significant finding was the elevation of Von Willebrand factor VIII antigen (> 150% activity, normal range 50–150, ELISA) as a result of the vasculitic process. Echo Doppler ultrasound of the upper and lower extremities excluded deep vascular thrombosis.

A diagnosis of adenovirus‐induced PF was made [1]. Due to the rapid progression of the lesions and the severity of the overall clinical picture, after the parents' consent, immunomodulatory therapy with high‐dose anakinra (8 mg/kg/day intravenously.), heparin (200 U/kg/day) and broad‐spectrum antibiotics was promptly started.

**FIGURE 2:**
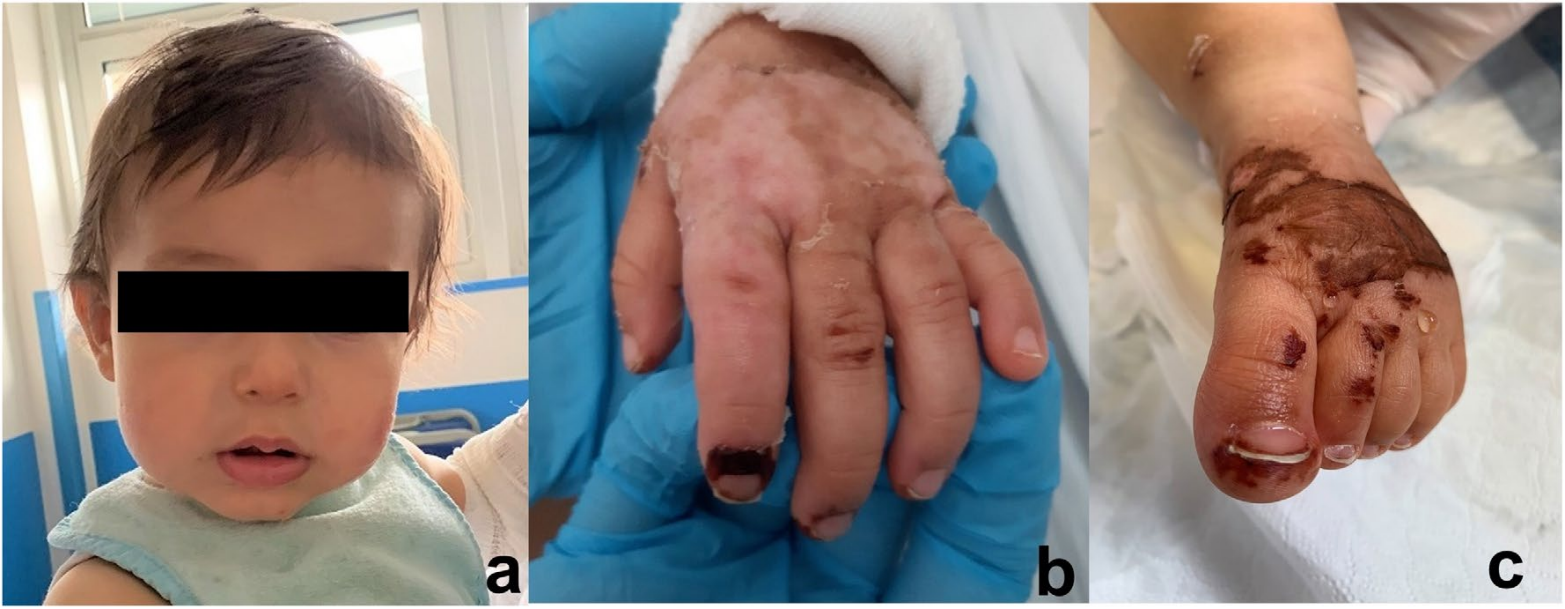
Clinical picture on Day 7: Disappearance of the face lesions (a), healing evolution of the purpuric lesions and regression of the fingertip cyanosis (b, c).

The finger pain stopped within 24 h and the cyanosis resolved within 48 h, followed by the hemorrhagic blisters in the following days (Figure 2a–c). The vital signs remained normal with no need for vasoactive drugs. With the gradual improvement of both skin lesions and laboratory tests, anticoagulation treatment was discontinued, and anakinra was tapered to 2 mg/kg/day on Day 7 and discontinued altogether on Day 21. In the 12‐month follow‐up period, the patient was in good general condition and remained symptom‐free with no sequelae or scarring.

## 3 Discussion

PF is a rare hematologic and dermatologic emergency, characterized by rapidly progressive purpura and necrosis of extensive areas of the skin, often associated with disseminated intravascular coagulation (DIC) [1, 3]. PF is characterized by a hypercoagulable state secondary to inflammation and endothelial damage. The rapid clinical progression correlates with histological findings consisting of extensive venous thrombosis of the dermis with hemorrhagic tissue infarction and leukocytoclastic vasculitis [1]. These histological and pathophysiological features clearly justify the two main aims of treatment: reduction of the hypercoagulable state and control of the inflammation‐related endothelial damage.

This was the rationale for choosing, in our emergency setting, the most suitable treatment for this patient. Given the important inflammatory state, in addition to antibiotic and anticoagulant therapy, the choice of anti‐inflammatory treatment was not easy. Corticosteroids, which are generally used in pediatric vasculitis, were not recommended in this case since they activate coagulation factor VIII with a predictable procoagulant effect [9].

Given the pivotal role of IL‐1 in the systemic and endothelial inflammatory state observed in several pediatric conditions [10, 11], we chose anakinra for its rapid IL‐1 blockade and excellent safety profile. As in other life‐threatening conditions, we used higher doses of anakinra until the inflammation was under control, then gradually tapered down [12]. This therapeutic strategy, adopted for a brief period of time, is not associated with increased side effects. IL‐1 is the main mediator of inflammation in vasculitis, as is true in other polyfactorial inflammatory diseases [13, 14]. In particular, there is evidence that supports the use of anakinra for the treatment of severe Henoch Schonlein purpura, a microangiopathic vasculitis similar to PF, and in Kawasaki disease, where IL‐1 plays a pivotal role in the endothelial inflammation [14, 15]. Indeed, the presence of a concomitant infection did not represent a major contraindication as anakinra showed efficacy in patients with severe sepsis and in COVID‐19 patients with respiratory distress syndrome [16, 17, 18].

Therefore, blocking the IL‐1 pathway was a valid therapeutic option in our young patient. The parents, aware of the high risk of a possible rapid progression of the disease with a thrombotic event and even limb amputation, gave their consent to such experimental treatment with anakinra.

In conclusion, it is important to consider PF in the differential diagnosis of critically ill children presenting rapidly evolving necrotizing lesions. To the best of our knowledge, this is the first report of a child with severe PF successfully treated with high‐dose anakinra. This case suggests that anakinra may be added to the existing treatment protocol of infectious or post‐infectious PF, including classical anticoagulants, protein‐C concentrate, and treatment of the underlying infection. Considering that the severe final outcome of PF is similar in both adults and children [19], this multifaceted approach might be extended to adult patients with rapidly evolving PF at high risk of developing deep thrombosis that frequently leads to limb amputation or even death.

## Author Contributions

F.Z. and A.M. are the senior authors. F.Z., L.S., and A.A. designed the study. F.T. and A.M. performed the systematic literature review. F.Z. wrote the first draft of the manuscript and all the other co‐authors revised and approved the final version of the manuscript.

## Ethics Statement

Ethical Committee approval was not needed because Anakinra is already in use with similar protocols in pediatric patients for other similar indications.

## Consent

Signed informed consent from the parents was obtained.

## Conflicts of Interest

The authors declare no conflicts of interest.

## Data Availability

The data that support the findings of this study are available from the corresponding author upon reasonable request.
